# Spatial neophobia is still not correlated with object neophobia in wild-caught house sparrows (*Passer domesticus*)

**DOI:** 10.1098/rsos.250220

**Published:** 2025-05-21

**Authors:** Blake A. Dusang, Marquise S. Henry, Melanie G. Kimball, Ella B. Cochran, Michael B. Wilson, Christine R. Lattin

**Affiliations:** ^1^ Biological Sciences, Louisiana State University, Baton Rouge, LA, USA

**Keywords:** behavioural syndromes, personality, temperament, coping styles, boldness, exploration

## Abstract

Neophobia, aversive behaviour towards novel objects, foods and environments, is a trait that affects the ability of animals to adapt to new environments and exploit novel resources. Our previous work demonstrated that individual responses of house sparrows (*Passer domesticus*) to object neophobia trials were not correlated with time spent in or latency to enter a novel environment. However, because no positive stimulus was present in the novel environment, this study may have measured spatial *neophilia*. In the present study, we placed familiar food dishes in a novel environment and assessed whether an individual’s willingness to enter and feed was significantly correlated with its willingness to feed from a familiar dish containing a novel object in the home cage. We exposed house sparrows (*n* = 26) to a novel environment and measured their latency to enter and feed, total time spent in the novel environment and total feeding time. Sparrows were also assessed for object neophobia in their home cage. Results indicated that there were no correlations between any of the measured behaviours in the novel environment and individual responses to novel object trials, suggesting that even with food as a common motivator, spatial neophobia and object neophobia represent two distinct traits.

## 1. Introduction

As natural environments worldwide are replaced with novel urban and suburban environments, the pressure on animals to consume novel foods, approach novel objects and explore new environments increases [1]. Neophobia, the avoidance of novel foods, objects and environments, is a widespread and often repeatable behaviour that directly influences an animal’s ability to respond to changes in available resources, especially in new or human-altered environments [2,3]. In fact, several studies have shown that neophobia may help explain why some species, populations or individuals persist in urbanized landscapes while others do not [1,4], and neophobia may also be a factor in invasion success [5,6].

Neophobia is usually measured in the laboratory or field by assessing an animal’s willingness to approach a positive stimulus like food or a nesting site when it is paired with something novel and potentially aversive [7]. Novel stimuli used to assess neophobia include objects, foods, environments, sounds, smells and tastes [8–12], and researchers often assume that an animal’s response to one type of novelty reflects its response to other types of novel stimuli (e.g. food neophobia correlating with object neophobia). However, responses to novelty are often context-specific and can be affected by factors such as sociality [13], season [14] and, in novel environments, habitat density [15]. Despite these context-specific effects, it is generally assumed that different types of neophobia tests demonstrate ‘convergent validity’ and reflect the same underlying stable personality trait: responses to novel objects or novel foods, for example, are considered to reflect an animal’s underlying neophobia, rather than its ‘object neophobia’ or ‘food neophobia’ [16].

This assumption of convergent validity has been tested in some studies, with somewhat mixed results. In particular, research comparing animals’ responses to novel spatial environments and novel objects sometimes shows positive correlations within individuals [17–20], but other studies find no relationship between novel object and novel spatial environment responses [21–24]. This variation suggests that these different tests may not be measuring the same underlying personality trait. However, there are also inconsistencies in how different studies were conducted that may explain some of this variation in results. For example, some studies placed animals into novel environments, whereas others gave animals a choice of whether to enter [21,25]; some spatial neophobia tests also exposed animals to novel objects like artificial trees [20], which could potentially confound object and spatial neophobia.

Another potential inconsistency is between studies that assessed neophilia (novelty attraction or preference) instead of neophobia (novelty avoidance). Some ‘neophobia’ studies have measured animals’ willingness to approach novel objects or enter a novel environment in the absence of a positive stimulus like food (e.g. [7,26]). However, in the absence of food or another motivator, the only reason for animals to approach is if they show an interest or even a preference for novel over familiar stimuli (i.e. neophilia) [8]. There is some evidence that neophobia and neophilia are distinct traits [27,28], and many wild species do not show neophilia, though factors such as urbanization and proximity to humans can affect whether a given population exhibits neophilia [29,30]. However, a preference for novel over familiar stimuli is common in domesticated animals like laboratory rodents and Japanese quail [31,32].

In this study, our goal was to determine if object neophobia was correlated with spatial neophobia in wild-caught house sparrows (*Passer domesticus*) in a laboratory setting. House sparrows exhibit large and repeatable variation in responses to novel foods, objects, environments and novel object habituation responses [10,33–35], and some individuals display high levels of neophobia while others appear indifferent towards novelty, making house sparrows an ideal organism to study intra-individual correlations among different kinds of neophobia tests [34,36]. A previous study evaluating house sparrow neophobia in the context of novel objects and novel environments found no correlation between individual responses to the two paradigms. However, no positive stimulus was present in the novel environment, so this study may have compared spatial neophilia to object neophobia [24]. By adding a familiar food dish to the novel environment as a positive stimulus, we sought to compare spatial neophobia to object neophobia. If spatial and object paradigms are measuring the same underlying personality trait [37], we expected an individual sparrow’s latency to enter a new environment and total time spent in the new environment would be significantly correlated with its latency to approach a novel object near food and feed from a dish containing a novel object.

## 2. Methods

### 2.1. Study subjects

Adult house sparrows (*n* = 13 females, *n* = 13 males) were captured using mist nets at bird feeders in East Baton Rouge Parish between April 2023 and January 2024. In a vivarium at Louisiana State University, sparrows were singly housed in sets of three identical cages separated by solid metal dividers and had unlimited access to mixed seeds, Mazuri small songbird diet (a vitamin-rich food supplement), grit and water. Sparrows were allowed to acclimate for at least three weeks before the start of experiments and were maintained at a day length of 13 L : 11 D during acclimation and trial periods. Animals were collected under Louisiana State Scientific Collecting Permits, and all procedures were approved by the Louisiana State University Institutional Animal Care and Use Committee under protocol no. 2021-010. We used approved methods for bird capture, transport and husbandry as specified in the Ornithological Council’s Guidelines to the Use of Wild Birds in Research [38]. After project completion, sparrows were used for further studies.

### 2.2. Spatial and object neophobia trials

Sparrows were visually isolated from each other during behaviour trials to prevent influence from conspecifics [33]. Three days prior to the beginning of trials, sparrows were moved to the centre cage of each three-cage set with solid metal dividers separating the empty left and right cages from the centre cage. Centre cages contained food and water dishes and three different types of perches: a plastic perch, a manzanita branch and latex tubing. Left and right cages were set up with the same objects placed in different locations to avoid conflating novel object and novel environment responses. Object and spatial neophobia trials took place on separate weeks. The first group of sparrows (*n* = 7 females, *n* = 4 males) was tested for object neophobia before spatial neophobia, while the second group of sparrows (*n* = 6 females, *n* = 9 males) underwent spatial neophobia trials prior to object neophobia trials. Note that previous neophobia studies in house sparrows found no difference in neophobia between sexes [24,33,36].

For all behaviour trials, sparrows were fasted overnight. The following morning, researchers entered the room 30 min after lights turned on and began video recordings. Spatial neophobia trials followed methods from Kimball & Lattin [7], except that during the spatial neophobia week, food was placed in the back centre of left and right cages for experimental trials (*n* = 2 for each sparrow), or in the centre cage for control trials (*n* = 2 for each sparrow). Researchers opened either the right or left cage divider approximately 5 cm or wiggled the divider for control trials, then left the room and recorded 1 h videos using pole-mounted security cameras (ZOSI Z18.5.T.2) located approximately 1.2 m from cages and connected to a DVR (ANNKE Model DM310). After 1 h, we stopped the video recordings, moved experimental trial birds and their food back to the centre cages and closed all dividers. Sparrows experienced four trials over 4 days consisting of two control trials and two experimental trials in a random order.

During the object neophobia week, sparrows randomly received three of seven possible novel objects presented with food and 2 days of control (food only; no object) trials. Object neophobia or control trials took place over five consecutive days in a random order. For object neophobia trials (*n* = 3 for each sparrow), food dishes without objects were replaced with dishes containing food, grit and a novel object (<10 cm^2^ in size) in, on, over or around the food dish. Novel objects used were white paper covers, foam fans, glitter stars, green drink umbrellas, gold jingle bells, brown Lego arches, multicoloured paperclip chains, pink puffs, yellow pipe cleaners, plastic purple eggs and red food dishes. All novel objects were previously piloted in different groups of sparrows [24,33,39] and found to significantly increase sparrows’ latency to feed compared to the food dish alone. Some sparrows had participated in prior neophobia studies involving some of the objects, so not all sparrows saw the same set of three objects to ensure that all the objects used were novel to each individual. For control trials (*n* = 2 for each sparrow), normal food dishes were returned to the cage. We recorded 1 h of behaviour and, after video recordings were stopped, we removed novel objects from food dishes.

### 2.3. Behaviour analyses

Behaviour was measured using BORIS 7.10.2 [40]; all spatial neophobia videos were scored by the same observer. Each spatial neophobia video was observed for an individual’s latency to enter the new environment, its latency to approach its food, its latency to eat its food, total duration spent in the new environment, total count of approaches to the entrance of the new environment before entering and total duration spent eating. Ethograms were created as previously described to associate different computer keys with state-type behaviours (table 1; [39]). Videos for object neophobia were observed for an individual’s latency to approach its food dish, as well as its latency to feed from the food dish. In previous studies, assessments of these behaviours have been highly consistent among different observers [6].

**Table 1.** Ethogram used for assessing house sparrow behaviours during spatial and object neophobia trials. (Each behaviour was assigned a different key using BORIS software.)

spatial neophobia		
key	code	description
*n*	latency to enter a new cage	time from the start of a trial for a sparrow to enter the new cage. Time was started at the beginning of trials after researchers exited the room and stopped when a sparrow crossed the divider with its whole body
*a*	latency to approach the food dish	time from the start of a trial for a sparrow to approach the food dish. Time was started when researchers exited the room and stopped when a sparrow was within one body length of the dish
*f*	latency to feed	time from the start of a trial for a sparrow to feed from the food dish. Time was started when researchers exited the room and stopped when a sparrow first lowered its head and pecked at its food
*d*	duration in the new cage	total time spent in the new cage. Time was started every time a sparrow entered the new cage and stopped every time the sparrow crossed the divider to return to its home cage
*e*	eating duration	total time spent eating. Time was started every time a sparrow first lowered its head and pecked at its food and stopped when the sparrow left the dish or stopped eating for more than 2 s
*t*	count of entrance approaches	total number of times the sparrow approached and was within one body length of the entrance to the novel environment before entering for the first time

object neophobia		
key	code	description
*a*	latency to approach the food dish	time from the start of a trial for a sparrow to approach the food dish. Time was started when researchers exited the room and stopped when a sparrow was within one body length of the dish
*f*	latency to feed	time from the start of a trial for a sparrow to feed from the food dish. Time was started when researchers exited the room and stopped when a sparrow first lowered its head and pecked at its food

### 2.4. Statistical analyses

All statistical analyses were conducted in R v. 4.3.3 [41]. Average responses for each sparrow across each trial type (i.e. the three object neophobia trials and the two spatial neophobia trials) were used to compute correlations. Neither novel environment nor novel object behavioural responses were normally distributed (Shapiro-Wilks tests, all *p* < 0.05). As a result, correlations between spatial and object neophobia were analysed using the ‘cor’ function in R to conduct Spearman’s rank correlation analyses. Effects of sex and trial number were investigated using Cox proportional hazard models through the ‘coxme’ function of the coxme package [42]. There was no effect of trial number (all *p* > 0.60, *z* > 0.14) or sex (all *p* > 0.12, *z* > 0.60) on spatial or object neophobia responses. Because of this, these variables were excluded from the final models. A Cox proportional hazard model was used to confirm that the novel objects used significantly increased sparrows’ latency to feed compared to the control condition. Another Cox proportional hazard model confirmed that placing food in either the left or right cage significantly increased latency to feed compared to the control cage. We created Kaplan-Meier survival curves using the ‘survfit’ function from the survival package [43] to visualize these data. ANOVA-based repeatability [44] of response to object and spatial trials were calculated using the ‘rpt.aov’ function from the ‘rptR’ package [45].

## 3. Results

**Figure 1.**
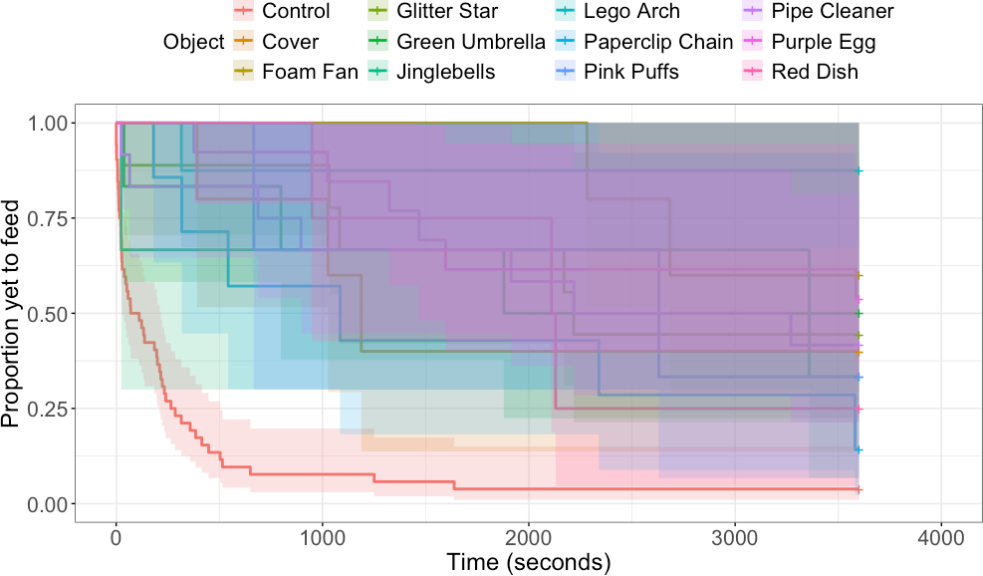
House sparrows (*n* = 26) took significantly longer to feed in the presence of novel objects presented with the food dish compared to the food dish alone (all *z* < −3.10, all *p* < 0.01). Average responses to control trials are represented by the red line and average responses to each novel object are represented by all other lines. The shaded areas represent 95% confidence interval.

**Figure 2.**
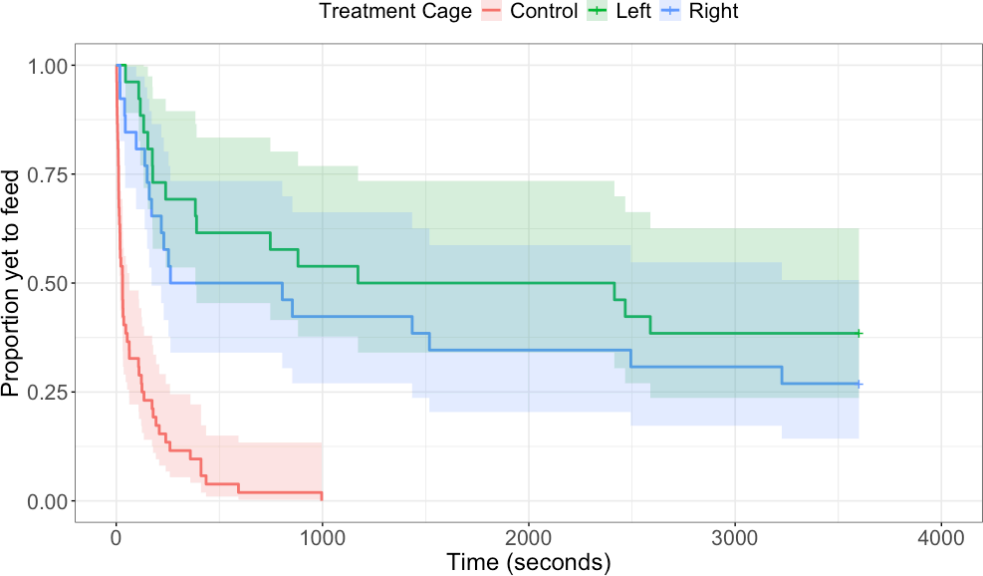
House sparrows (*n* = 26) took significantly longer to feed from a familiar food dish in a novel cage compared to the home cage (all *z* < −5.45, all *p* < 0.01). Control trials are represented by the red line, and novel cages are represented by blue and green lines. The shaded areas represent 95% confidence interval.

During the week of object neophobia trials, house sparrows’ feeding latency in the presence of all novel objects was significantly longer compared to control trials (all *z* < −3.10, all *p* < 0.01; figure 1), as was approach latency (all *z* < −2.7, all *p* < 0.01). House sparrows took longer to feed in the presence of the Lego arch compared to the paperclip chain (*z* = −2.36, *p* = 0.018). There were no other differences in feeding latency between different novel objects. During the week of spatial neophobia trials, there was no effect of left versus right side on sparrows’ latency to enter a novel environment in the presence of food (*z* = 0.47, *p* = 0.72; figure 2) or their latency to feed in a novel environment (*z* = 1.7, *p* = 0.088). For object neophobia, sparrows’ average latency to approach a novel object was significantly correlated with average latency to feed in the presence of a novel object (*r* = 0.95, *p* < 0.0001; table 2). For spatial neophobia, sparrows’ average latency to enter a novel environment was correlated with their average latency to feed in a novel environment (*r* = 0.73, *p* < 0.0001; table 2). However, there were no significant correlations between any object and spatial neophobia measures (all *r* < 0.4, all *p* > 0.1; table 2). Individual latency to feed in the two spatial neophobia trials (*r* = 0.67, *p* < 0.001) was slightly more repeatable than during the three object neophobia trials (*r* = 0.62, *p* < 0.001).

**Table 2.** While different measures of neophobia were correlated within each test type (e.g. latency to approach a novel object with latency to feed in the presence of a novel object, two different measures from object neophobia trials), there were no correlations between average object and spatial neophobia measures in house sparrows (*n* = 26). (Significant relationships are in bold. Top numbers are *p*-values and bottom numbers are Spearman’s correlation coefficients. See table 1 for details on behaviours analysed.)

	spatial entrance latency	spatial approach latency	spatial feed latency	spatial time in novel cage	spatial time feeding	count of entrance approaches	object approach latency	object feed latency
spatial entrance latency	—	**<0.0001** (***r* = 0.79**)	**<0.001** (***r* = 0.68**)	**<0.0001** (***r* = −0.71**)	**<0.0001** (***r* = −0.73**)	0.27 (*r* = 0.22)	0.18 (*r* = 0.27)	0.40 (*r* = 0.17)
spatial approach latency	—	—	**<0.0001** (***r* = 0.87**)	**0.01** (***r* = −0.49**)	**<0.0001** (***r* = −0.73**)	0.74 (*r* = 0.07)	0.32 (*r* = 0.20)	0.22 (*r* = 0.25)
spatial feed latency	—	—	—	**>0.01** (***r* = −0.53**)	**<0.0001** (***r* = −0.7**)	0.77 (*r* = 0.06)	0.21 (*r* = 0.26)	0.094 (*r* = 0.33)
spatial time in novel cage	—	—	—	—	**<0.0001** (***r* = 0.72**)	0.27 (*r* = 0.23)	0.76 (*r* = −0.06)	0.96 (*r* = 0.01)
spatial time feeding	—	—	—	—	—	0.45 (*r* = −0.15)	0.36 (*r* = −0.19)	0.35 (*r* = −0.19)
count of entrance approaches	—	—	—	—	—	—	0.20 (*r* = 0.26)	0.33 (*r* = 0.20)
object approach latency	—	—	—	—	—	—	—	**<0.0001** (***r* = 0.95**)

## 4. Discussion

Our objective was to determine whether house sparrows’ responses to a novel environment were correlated with their responses to novel objects when motivation was standardized. By adding a familiar food dish into the novel environment, we introduced a positive stimulus to reduce the risk of measuring spatial neophilia (an interest in or desire to explore a novel environment) and to standardize motivation across object and spatial neophobia testing paradigms [46]. We predicted that house sparrows which were neophobic towards novel objects near their food would exhibit the same cautious behaviour when presented with familiar food in a novel environment. However, we found no correlation between sparrows’ novel object and novel environment responses, suggesting that these two tests do not show convergent validity and that they may measure two different traits [16]. Some studies that have compared novel object and novel environment responses within individuals found a similar lack of correlation [22,24], while others found correlated responses to the two paradigms [17,19,47].

Inconsistent results across different species and studies may partly result from differences in experimental design, such as how animals are introduced to the novel environment—either by forced entry [19,25] or by allowing for choice of entry [24,48]—which could affect whether animals show exploration as compared to escape responses. There could also be between-study differences in the specific outcomes assessed. There is no set standard for how to measure the exploratory behaviour of wild animals in spatial neophobia tests, but behaviours such as latency to enter the novel environment and total time spent in the novel environment are often quantified [49]. Indeed, we found that most of the measures we used to assess both spatial and object neophobia were correlated within each context (e.g. time to enter a novel cage with time spent feeding in a novel cage).

Different testing paradigms may produce correlated results because the tests show convergent validity (they measure the same underlying trait) or because they measure two different traits that are correlated as part of a behavioural syndrome [50]. Behavioural syndromes among distinct personality traits such as aggression and activity are found in some species and studies, but not in others [51–53]. Therefore, it is also possible that some species show a behavioural syndrome that includes both spatial and object neophobia, while others do not. However, meta-analysis approaches have found that most correlated behaviours show small effect sizes, implying that very large sample sizes would be necessary to find the (true) correlations between these different behaviours [54,55]. Therefore, it is also possible that our sample size and resulting statistical power were not sufficient to find a behavioural syndrome between object neophobia and food neophobia that may actually exist in house sparrows.

We used access to food after an overnight fast as a motivating stimulus. Without a way to standardize motivation across different testing paradigms, it is difficult to determine whether tests measure the same trait or two different traits. However, while the food placed in the novel environment was intended to standardize motivation, there can also be individual variation in food motivation, or in responses to other motivating stimuli, that could cause different results across different tests [56].

Further consideration of sparrows’ behaviours in this study led us to question whether the spatial neophobia test might even be measuring different traits in different individual sparrows. Some birds fed from the dish in the novel environment immediately upon entrance. For these birds, the test may have been measuring food motivation versus fear of the new environment, a kind of approach-avoidance behaviour [57]. Alternatively, for birds that immediately entered the novel environment but never fed from the dish, this test may have measured escape or exploration behaviour. Exploration behaviour is sometimes assessed as whether animals will choose a novel stimulus over a rewarded one [58]. However, it is difficult for an outside observer to distinguish between intrinsic exploration versus escape behaviours [59]. For example, in this study, the behaviour of the birds that entered the novel cage but did not eat could be interpreted as exploration (choosing to explore the novel environment rather than approach the food) or as escape.

Even after introducing food as a common motivating factor, object neophobia was not correlated with spatial neophobia in house sparrows. Our results suggest that the tests used to evaluate object neophobia and spatial neophobia are not measuring the same underlying trait, although they may still be linked by a behavioural syndrome that we did not have the sample size to detect. Although we attempted to standardize motivation across paradigms, the behaviours of sparrows to novel objects did not predict their responses to the novel environment. It would be useful to examine measures of stress or anxiety such as circulating corticosterone or neuronal activity in brain regions like the medial ventral arcopallium (thought to be analogous to the mammalian amygdala [60]) in birds that immediately entered novel cages but did not feed (potential escapees/explorers) compared to those that did not enter the novel cage at all or those that entered and immediately fed. This paradigm could help distinguish between sparrows that enter the novel environment with the intention of exploring and those that are motivated by food to enter. However, using this particular spatial neophobia test to investigate ‘neophobia’ in house sparrows is probably not suitable, as we cannot definitively distinguish between whether individuals showed exploratory, escape, or neophobic behaviours.

## Data Availability

The authors confirm that the data supporting the findings of this study are available within this article's electronic supplementary material [41]. Supplementary material is available online [61].

## References

[1] Greggor AL, Thornton A, Clayton NS. 2015 Neophobia is not only avoidance: improving neophobia tests by combining cognition and ecology. Curr. Opin. Behav. Sci. **6**, 82–89. (10.1016/j.cobeha.2015.10.007)

[2] Greenberg R, Mettke-Hofmann C. 2001 Ecological aspects of neophobia and neophilia in birds. In Current ornithology, vol. 16 (eds V Nolan, CF Thompson), pp. 119–178. Boston, MA: Springer US. (10.1007/978-1-4615-1211-0_3)

[3] Modlinska K, Stryjek R. 2016 Food neophobia in wild rats (Rattus norvegicus) inhabiting a changeable environment—a field study. PLoS ONE **11**, e0156741. (10.1371/journal.pone.0156741) 27254150 PMC4890768

[4] Magory Cohen T, Kumar RS, Nair M, Hauber ME, Dor R. 2020 Innovation and decreased neophobia drive invasion success in a widespread avian invader. Anim. Behav. **163**, 61–72. (10.1016/j.anbehav.2020.02.012)

[5] Damas-Moreira I, Riley JL, Harris DJ, Whiting MJ. 2019 Can behaviour explain invasion success? A comparison between sympatric invasive and native lizards. Anim. Behav. **151**, 195–202. (10.1016/j.anbehav.2019.03.008)

[6] Krajcir KJ *et al*. 2024 Eurasian tree sparrows are more food neophobic and habituate to novel objects more slowly than house sparrows. Biol. Invasions **26**, 3677–3693. (10.1007/s10530-024-03403-5)

[7] Kimball MG, Lattin CR. 2024 The ‘seven deadly sins’ of neophobia experimental design. Integr. Comp. Biol. **64**, 38–54. (10.1093/icb/icad127) 37996398

[8] Greenberg R. 1983 The role of neophobia in determining the degree of foraging specialization in some migrant warblers. Am. Nat. **122**, 444–453. (10.1086/284148)

[9] Liebl AL, Martin LB. 2012 Exploratory behaviour and stressor hyper-responsiveness facilitate range expansion of an introduced songbird. Proc. R. Soc. B **279**, 4375–4381. (10.1098/rspb.2012.1606) PMC3479806 22951742

[10] Martin LB, Fitzgerald L. 2005 A taste for novelty in invading house sparrows, Passer domesticus. Behav. Ecol. **16**, 702–707. (10.1093/beheco/ari044)

[11] McCormick MI, Chivers DP, Allan BJM, Ferrari MCO. 2017 Habitat degradation disrupts neophobia in juvenile coral reef fish. Glob. Chang. Biol. **23**, 719–727. (10.1111/gcb.13393) 27393344

[12] Walsh S, Goulet CT, Wong BBM, Chapple DG. 2018 Inherent behavioural traits enable a widespread lizard to cope with urban life. J. Zool. **306**, 189–196. (10.1111/jzo.12582)

[13] Miller R *et al*. 2022 Socio-ecological correlates of neophobia in corvids. Curr. Biol. **32**, 74–85.(10.1016/j.cub.2021.10.045) 34793696

[14] Greggor AL, McIvor GE, Clayton NS, Thornton A. 2016 Contagious risk taking: social information and context influence wild jackdaws’ responses to novelty and risk. Sci. Rep. **6**, 27764. (10.1038/srep27764) 27282438 PMC4901300

[15] Mettke-Hofmann C, Eccles GR, Greggor AL, Bethell EJ. 2020 Cognition in a changing world: red-headed Gouldian finches enter spatially unfamiliar habitats more readily than do black-headed birds. Front. Ecol. Evol. **8**, 498347. (10.3389/fevo.2020.498347)

[16] Carter AJ, Feeney WE, Marshall HH, Cowlishaw G, Heinsohn R. 2013 Animal personality: what are behavioural ecologists measuring? Biol. Rev. **88**, 465–475. (10.1111/brv.12007) 23253069

[17] Cole EF, Quinn JL. 2014 Shy birds play it safe: personality in captivity predicts risk responsiveness during reproduction in the wild. Biol. Lett. **10**, 20140178. (10.1098/rsbl.2014.0178) 24829251 PMC4046374

[18] Martins CIM, Schaedelin FC, Mann M, Blum C, Mandl I, Urban D, Grill J, Schößwender J, Wagner RH. 2012 Exploring novelty: a component trait of behavioural syndromes in a colonial fish. Behaviour **149**, 215–231. (10.1163/156853912x634430) 31031407 PMC6485498

[19] Schuett W, Laaksonen J, Laaksonen T. 2012 Prospecting at conspecific nests and exploration in a novel environment are associated with reproductive success in the jackdaw. Behav. Ecol. Sociobiol. **66**, 1341–1350. (10.1007/s00265-012-1389-1)

[20] Verbeek MEM, Drent PJ, Wiepkema PR. 1994 Consistent individual differences in early exploratory behaviour of male great tits. Anim. Behav. **48**, 1113–1121. (10.1006/anbe.1994.1344)

[21] Fox RA, Millam JR. 2007 Novelty and individual differences influence neophobia in orange-winged Amazon parrots (Amazona amazonica). Appl. Anim. Behav. Sci. **104**, 107–115. (10.1016/j.applanim.2006.04.033)

[22] Martins TLF, Roberts ML, Giblin I, Huxham R, Evans MR. 2007 Speed of exploration and risk-taking behavior are linked to corticosterone titres in zebra finches. Horm. Behav. **52**, 445–453. (10.1016/j.yhbeh.2007.06.007) 17678929

[23] Ruuskanen S, Laaksonen T. 2010 Yolk hormones have sex-specific long-term effects on behavior in the pied flycatcher (Ficedula hypoleuca). Horm. Behav. **57**, 119–127. (10.1016/j.yhbeh.2009.09.017) 19804778

[24] Kimball MG, Lattin CR. 2023 Exploration of a novel environment is not correlated with object neophobia in wild-caught house sparrows (Passer domesticus). Behav. Processes **210**, 104913. (10.1016/j.beproc.2023.104913) 37406866

[25] Boogert NJ, Reader SM, Laland KN. 2006 The relation between social rank, neophobia and individual learning in starlings. Anim. Behav. **72**, 1229–1239. (10.1016/j.anbehav.2006.02.021)

[26] Quesada J, Chávez–Zichinelli CA, García–Arroyo M, Yeh PJ, Guevara R, Izquierdp-Palma J, MacGregor-Fors I. 2022 Bold or shy? Examining the risk–taking behavior and neophobia of invasive and non–invasive house sparrows. Anim. Biodiv. Conserv. **45**, 97–106. (10.32800/abc.2022.45.0097)

[27] Mettke‐Hofmann C, Winkler H, Leisler B. 2002 The significance of ecological factors for exploration and neophobia in parrots. Ethology **108**, 249–272. (10.1046/j.1439-0310.2002.00773.x)

[28] Miranda AC, Schielzeth H, Sonntag T, Partecke J. 2013 Urbanization and its effects on personality traits: a result of microevolution or phenotypic plasticity? Glob. Chang. Biol. **19**, 2634–2644. (10.1111/gcb.12258) 23681984

[29] McLean LRW, Goodman TF, Horton TW, Nelson XJ. 2024 Effects of proximity to humans on neophilia, foraging ecology and population structure of kea. N. Z. J. Zool. **51**, 258–274. (10.1080/03014223.2023.2274838)

[30] Tryjanowski P *et al*. 2016 Urbanization affects neophilia and risk-taking at bird-feeders. Sci. Rep. **6**, 28575. (10.1038/srep28575) 27346383 PMC4921825

[31] Kiyokawa Y, Ootaki M, Kambe Y, Tanaka KD, Kimura G, Tanikawa T, Takeuchi Y. 2024 Approach/avoidance behavior to novel objects is correlated with the serotonergic and dopaminergic systems in the brown rat (Rattus norvegicus). Neuroscience **549**, 110–120. (10.1016/j.neuroscience.2024.05.003) 38723837

[32] Damphousse CC, Miller N, Marrone DF. 2022 Dissociation of spatial and object memory in the hippocampal formation of Japanese quail. iScience **25**, 103805. (10.1016/j.isci.2022.103805) 35243216 PMC8859546

[33] Kelly TR, Kimball MG, Stansberry KR, Lattin CR. 2020 No, you go first: phenotype and social context affect house sparrow neophobia. Biol. Lett. **16**, 20200286. (10.1098/rsbl.2020.0286) 32871090 PMC7532720

[34] McLaughlin AL, Westneat DF. 2023 House sparrows exhibit individual differences in generalization when confronted with different novel stimuli. Ethology **129**, 369–379. (10.1111/eth.13374)

[35] Tuliozi B, Fracasso G, Hoi H, Griggio M. 2018 House sparrows’ (Passer domesticus) behaviour in a novel environment is modulated by social context and familiarity in a sex-specific manner. Front. Zool. **15**, 16. (10.1186/s12983-018-0267-8) 29721031 PMC5910580

[36] Ensminger AL, Westneat DF. 2012 Individual and sex differences in habituation and neophobia in house sparrows (Passer domesticus). Ethology **118**, 1085–1095. (10.1111/eth.12009)

[37] Réale D, Reader SM, Sol D, McDougall PT, Dingemanse NJ. 2007 Integrating animal temperament within ecology and evolution. Biol. Rev. **82**, 291–318. (10.1111/j.1469-185x.2007.00010.x) 17437562

[38] Fair J, Paul E, Jones J, Bies L (eds). 2023 Guidelines to the use of wild birds in research. Washington, D.C: Ornithological Council.

[39] Kimball MG, Lattin CR. 2023 Exploration of a novel environment is not correlated with object neophobia in wild-caught house sparrows (Passer domesticus). Behav. Process. **210**, 104913. (10.1016/j.beproc.2023.104913) 37406866

[40] Friard O, Gamba M. 2016 BORIS: a free, versatile open‐source event‐logging software for video/audio coding and live observations. Methods Ecol. Evol. **7**, 1325–1330. (10.1111/2041-210X.12584)

[41] R Core Team. 2024 R: a language and environment for statistical computing. Vienna, Austria: R Foundation for Statistical Computing. See https://www.R-project.org.

[42] Therneau TM. 2020 *coxme: mixed effects Cox models*. R-package. See https://CRAN.R-project.org/web/packages/coxme.

[43] Therneau TM. 2021 *survival: a package for survival analysis in R*. R package. See https://CRAN.R-project.org/package=survival.

[44] Lessells CM, Boag PT. 1987 Unrepeatable repeatabilities: a common mistake. Auk **104**, 116–121. (10.2307/4087240)

[45] Stoffel MA, Nakagawa S, Schielzeth H. 2017 rptR: repeatability estimation and variance decomposition by generalized linear mixed‐effects models. Methods Ecol. Evol. **8**, 1639–1644. (10.1111/2041-210x.12797)

[46] Mettke‐Hofmann C, Lorentzen S, Schlicht E, Schneider J, Werner F. 2009 Spatial neophilia and spatial neophobia in resident and migratory warblers (Sylvia). Ethology **115**, 482–492. (10.1111/j.1439-0310.2009.01632.x)

[47] Butler MW, Toomey MB, McGraw KJ, Rowe M. 2012 Ontogenetic immune challenges shape adult personality in mallard ducks. Proc. R. Soc. B **279**, 326–333. (10.1098/rspb.2011.0842) PMC3223679 21653587

[48] Schuett W, Godin JGJ, Dall SRX. 2011 Do female zebra finches, Taeniopygia guttata, choose their mates based on their ‘personality’? Ethology **117**, 908–917. (10.1111/j.1439-0310.2011.01945.x)

[49] Huang P, Kerman K, Sieving KE, St. Mary CM. 2016 Evaluating the novel-environment test for measurement of exploration by bird species. J. Ethol. **34**, 45–51. (10.1007/s10164-015-0444-6)

[50] Sih A, Bell AM, Johnson JC, Ziemba RE. 2004 Behavioral syndromes: an integrative overview. Q. Rev. Biol. **79**, 241–277. (10.1086/422893) 15529965

[51] Garamszegi LZ, Markó G, Szász E, Zsebők S, Azcárate M, Herczeg G, Török J. 2015 Among-year variation in the repeatability, within- and between-individual, and phenotypic correlations of behaviors in a natural population. Behav. Ecol. Sociobiol. **69**, 2005–2017. (10.1007/s00265-015-2012-z) 26586925 PMC4642588

[52] Kortet R, Hedrick A. 2007 A behavioural syndrome in the field cricket Gryllus integer: intrasexual aggression is correlated with activity in a novel environment. Biol. J. Linn. Soc. **91**, 475–482. (10.1111/j.1095-8312.2007.00812.x)

[53] Wilson ADM, Whattam EM, Bennett R, Visanuvimol L, Lauzon C, Bertram SM. 2010 Behavioral correlations across activity, mating, exploration, aggression, and antipredator contexts in the European house cricket, Acheta domesticus. Behav. Ecol. Sociobiol. **64**, 703–715. (10.1007/s00265-009-0888-1)

[54] Garamszegi LZ, Markó G, Herczeg G. 2013 A meta-analysis of correlated behaviors with implications for behavioral syndromes: relationships between particular behavioral traits. Behav. Ecol. **24**, 1068–1080. (10.1093/beheco/art033)

[55] Garamszegi LZ, Markó G, Herczeg G. 2012 A meta-analysis of correlated behaviours with implications for behavioural syndromes: mean effect size, publication bias, phylogenetic effects and the role of mediator variables. Evol. Ecol. **26**, 1213–1235. (10.1007/s10682-012-9589-8)

[56] Barnard CJ, Sibly RM. 1981 Producers and scroungers: a general model and its application to captive flocks of house sparrows. Anim. Behav. **29**, 543–550. (10.1016/s0003-3472(81)80117-0)

[57] O’Neil EB, Newsome RN, Li IHN, Thavabalasingam S, Ito R, Lee ACH. 2015 Examining the role of the human hippocampus in approach–avoidance decision making using a novel conflict paradigm and multivariate functional magnetic resonance imaging. J. Neurosci. **35**, 15039–15049. (10.1523/jneurosci.1915-15.2015) 26558775 PMC6605357

[58] O’Hara M, Mioduszewska B, von Bayern A, Auersperg A, Bugnyar T, Wilkinson A, Huber L, Gajdon GK. 2017 The temporal dependence of exploration on neotic style in birds. Sci. Rep. **7**, 4742. (10.1038/s41598-017-04751-0) 28684773 PMC5500574

[59] Hughes RN. 1997 Intrinsic exploration in animals: motives and measurement. Behav. Process. **41**, 213–226. (10.1016/s0376-6357(97)00055-7) 24896854

[60] Mello CV, Kaser T, Buckner AA, Wirthlin M, Lovell PV. 2019 Molecular architecture of the zebra finch arcopallium. J. Comp. Neurol. **527**, 2512–2556. (10.1002/cne.24688) 30919954 PMC6879308

[61] Dusang B, Henry M, Kimball MG, Cochran EB, Lattin CR, Wilson MBB. 2025. Supplementary Material from: Spatial Neophobia Is Still Not Correlated with Object Neophobia in Wild-Caught House Sparrows (Passer Domesticus). FigShare. (10.6084/m9.figshare.c.7811563) PMC12092101 40400518

